# Supplementary material for: Are carbon emissions trading and green financial instruments synergistic? -Comprehensive quantitative research based on content analysis
Source: PLoS One. 2024 Mar 7;19(3):e0298601. doi: 10.1371/journal.pone.0298601 (PMC10919601; doi:10.1371/journal.pone.0298601)
Supplement: S1 Data — (ZIP) [file pone.0298601.s001.zip › Data/地级市Z_F工作报告.zip!/─■╧─/╥°┤¿╩╨/└┤╫╘╣1⁄2╓┌║┼í╛┬φ┐╦╩2╛▌═°í┐_2014.docx]

各位代表：

　　现在，我代表市人民政府向大会作政府工作报告，请予审议，并请政协委员和其他列席人员提出意见。

　　一、2013年工作回顾

　　2013年是我们面对严峻复杂的国内外经济形势，推进大开放、大建设、大发展的一年。一年来，全市上下紧紧围绕加快建设“两个最适宜”城市、率先建成全面小康社会的目标，大力实施“2258”工作思路，抢抓机遇，迎难而上，务实苦干，较好地完成了市十四届人大一次会议确定的各项目标任务，经济社会发展稳中有进、稳中向好，实现了本届政府的良好开局。全年完成地区生产总值1273.49亿元，同比增长10%，其中一、二、三产业分别增长3.8%、11.8%和8.3%；地方公共财政预算收入134.6亿元，增长19%；全社会固定资产投资1149亿元，增长25.1%；社会消费品零售总额348.06亿元，增长12.2%；城镇居民人均可支配收入23781元，增长10%；农民人均纯收入9036元，增长12%。推动银川科学跨越发展取得新成就。

　　——项目带动成效显著。坚持经济工作项目化，深入开展“准备之冬”、“建设之春”、“发展之夏”和“收获之秋”系列活动，实施重点项目582个，完成投资867.3亿元，助推产业升级。

　　工业经济回升向好。积极应对年初工业经济低速增长的不利局面，加强运行调控和协调服务，及时出台《关于促进工业经济增长的若干意见》、《关于加快发展非公有制经济的实施意见》等政策措施，累计投入各类工业发展扶持资金3亿多元，帮助企业渡过难关。实施重点工业项目240个，神华宁煤400万吨煤制油、宝丰260万吨焦化二期、中石油宁夏石化“45/80”大化肥等重大项目进展顺利，工业增速逐月回升，完成规模以上工业增加值492.16亿元、增长12.4%。新能源、新材料、高端装备制造、电子信息等战略性新兴产业加快发展。银川经济技术开发区转型升级迈出新步伐，iBi育成中心一期建成投用，入驻企业达到120家，高新技术产业聚集效应初步显现，被评为“全国十佳最具投资竞争力园区”。宁夏生态纺织产业示范园“一园两区”建设加快推进，贺兰园区引进山东如意集团投资74亿元建设如意科技时尚产业城，建设厂房、仓库68.2万平方米；灵武园区基础设施基本完工，建设厂房60万平方米，成功创建国家级羊绒出口产品质量安全示范区。银川科技园基础设施基本完成，引进中科院建设西北唯一的科技创新与产业育成中心，宝塔石化、永成生物等企业研发中心入园建设。全年实施节能技改重点项目34个，新认定自治区级企业技术中心5个，神华宁煤、隆基宁光通过国家级企业技术中心验收。恒源万福、天人和等13家中小企业在天交所成功挂牌上市。工业运行质量和效益明显好转。

　　现代农业提质增效。“三精”农业加快发展，完成农业总产值103.95亿元、增长4%。新建、改造现代农业示范园区（基地）30个，农业科技贡献率达57%，贺兰县成为全国21个现代农业改革发展示范区之一。设施园艺总量达到33万亩，奶牛存栏16.2万头，粮食生产实现“十连增”。有机水稻、酿酒葡萄、供外蔬菜、万亩生态牧场等特色种养基地规模扩大、效益提高。国家、自治区级农业产业化龙头企业达到89家，创建农民专业合作示范社36个，培育家庭农场101家，流转土地10万亩，带动农户10.15万户。建设高标准农田81万亩，新增节水灌溉面积26.84万亩。完成“三品一标”认证认定429个，创建中国驰名商标、名牌产品9个，农产品质量安全水平不断提高。

　　服务业提速升级。区域商贸物流中心建设成效明显，“双十工程”进展迅速，新华联广场、永泰城、大阅城等商业综合体陆续开工，亘元万豪等星级酒店主体封顶，万达嘉华、悦海新天地、奥特莱斯名品折扣城开业运营；“大物流”格局初步形成，海尔（银川）虚实网服务园落户国际公铁物流中心，润恒农产品物流园、陆港物流中心等项目加快推进，全年实现社会物流总额2431亿元，增长10%；电子商务示范城市建设有序推进，淘宝网特色中国宁夏馆建成运营，跨境贸易电子商务试点获批。金融服务业加快发展，引进中信、浦发等金融机构，人民币存贷款余额分别增长11%和16.5%。会展经济初具规模，成功举办大型展会17场，参展人数超过100万人次。东西两条文化旅游带功能不断完善，华夏河图、韩美林艺术馆等项目加快推进，黄河军事文化博览园开园运营，成功举办首届西北风情自驾游等大型活动，全年接待游客707万人次，实现旅游收入70亿元、增长20%。

　　——开放步伐全面加快。滨河新区建设迅速起步。确立“生态城、文化城、旅游城、产业城”定位，编制完成战略规划、总体规划，收储土地12万亩。重点项目“百日大会战”成效突出，滨河工业园基础设施配套趋于完善，横城小镇一期、滨河中学等8个项目完工，国际医疗城、滨河生态文化园等25个重点项目全面开工，累计完成投资30多亿元，新区产业集聚、生态效应逐步显现。银川综合保税区封关运营。创造了在全国审批速度最快、建设速度最快的记录。引进领鲜·普诺斯保税仓储中心、中银绒业保税仓库、西部黄金珠宝文化产业园等21个项目，同步规划建设临港经济区等配套园区。阅海湾中央商务区初具雏形。“三园一轴”基础设施建成投用，银川国际交流中心全面启用，引进中石油宁夏分公司等总部项目39个。开放合作成效显著。对阿、对东北亚、对台开放不断拓展，成功承办举办中国（宁夏）韩国友好交流周、全国台企联银川行等重大活动，中俄韩医疗合作结出硕果，银川卡瓦心脏中心正式运营。开通银川至香港、台北、曼谷等客运航线，与法国博朗斯市、德国奥尔登堡市等4个城市缔结国际友好城市。全年实施招商引资项目290个，引进海尔、中铝等世界和中国500强企业5家，实际到位资金412.6亿元。实现外贸进出口总额24亿美元、增长76.4%，对外开放和国际化迈出了新步伐。

　　——城乡环境明显改善。坚持精品建设、精美打造、精细管理，“美丽银川”系列行动取得丰硕成果，获得中国人居环境奖。确立“全域银川”理念，启动城市长远发展战略规划研究。以道路畅通为重点的旧城（棚户区）改造顺利推进，累计拆迁改造1661.86万平方米，开工建设安置区34个、737.95万平方米，实现了和谐拆迁、群众受益。打通新建新华西街、湖滨西街、庆丰街等道路39条，改造小街巷13条，开工建设滨河、永宁黄河大桥及连接线工程。快速公交一号线稳定运营，公交分担率提高到26.8%。全力推进主干道大整治大绿化、滨河新区大环境绿化、唐徕公园六期整治、七子连湖扩整、西部水资源综合利用等一批重点生态工程，立法保护滨河新区黄河两侧、爱伊河沿线湿地，完成绿化面积6102公顷，湿地面积达到5.31万公顷，获得全国首批水生态文明试点城市。“蓝天工程”启动实施，药企污染、燃煤锅炉、机动车尾气等重点污染源治理初见成效，高标准完成永二干沟综合整治及徕龙公园建设，使昔日的“龙须沟”成为城市景观公园。全年空气质量优良天数达到252天，PM2.5达标率81%。实施城市精细化管理和文明细节行动，建立“大城管”体制，成立城市综合管理委员会、环境和食品药品公安分局及城管流动速裁法庭。“数字城管”建成运行，建立城市综合管理考核长效机制，机械化清扫保洁、渣土车整治、“中国式过马路”等专项治理卓有成效，顺利通过全国文明城市复检。城镇化和新农村建设步伐加快，德胜、望远片区纳入主城区统一规划，掌政镇、良田镇等6个特色小城镇建设初具规模，永宁八渠、贺兰兰光等中心村建设快速推进，新建21个幸福村庄示范点，完成农村环境集中连片整治10个，建成农村公路269公里，城乡面貌明显改观。

　　——民生事业有力推进。为民办10件实事全面完成，民生社会事业投入达178亿元，占公共财政支出的80%以上。出台支持青年大学生等重点群体创业优惠政策，建成全区首个大学生创业孵化基地，城镇新增就业6.3万人，城镇登记失业率为3.51%。社会保险扩面提标，提高企业退休人员基本养老金、城乡居民基础养老金和城乡低保标准，建立重特大疾病救助机制。新建社区老年人日间照料中心10个、居家养老服务站25个，建设市妇女儿童活动中心、残疾人康复中心。调整扩大住房保障范围，新建保障性安居工程8111套，改造城市棚户区3458套，4952户困难家庭享受住房保障，成为全国首批利用住房公积金支持保障性住房建设的试点城市。着力平抑物价，新建标准化菜市场10个、社区蔬菜直销店33个，加大肉、菜等食品价格调控力度，居民消费品价格指数控制在3.5%以内。生态移民、扶贫攻坚扎实推进，新建移民住房14817套，搬迁移民9361户、40590人，探索工厂建在村头等产业扶贫新模式，全力打造闽宁协作移民扶贫示范镇，生态移民地区人均纯收入达到5000元，同比增长30%左右。优质教育扩面提升工程启动实施，每年安排5000万元用于教育教学质量考核奖励，组建五大教育发展共同体，与北方民族大学共建附属中学，贺兰县、灵武市通过国家义务教育均衡发展县验收，成功创建2所全国职业教育改革发展示范校。医疗卫生服务水平不断提升，新建社区卫生服务站10所、标准化村卫生室33所，在全国率先试点推行“先住院、后付费”诊疗模式。低生育水平持续稳定。成功创建国家首批公共文化服务体系示范区，圆满举办亚洲首届IBHF世界杯马术绕桶赛、环青海湖国际公路自行车银川赛段等高规格赛事。加强和创新社会管理，银川信息网建设取得重大进展，进入“国家智慧城市试点”行列。平安创建工作不断深化，社会治安持续稳定，安全生产形势保持平稳，征地拆迁等信访突出问题得到有效化解，荣获“2009~2012年度全国社会管理综合治理优秀市”。民族团结进步创建活动扎实开展，国防动员、双拥工作稳步推进，防灾减灾、气象、统计、仲裁、档案、地方志等工作取得新成绩。

　　——政府效能不断提高。以“三减两提高”为核心的行政审批制度改革加快推进，全年共取消下放市级行政审批事项18项，减少审批程序61项，减少审批时限808个工作日，取消免征行政事业性收费26项，在全区率先建成四级政务服务网络，成为西北地区审批事项最少、速度最快的城市。主动接受人大依法监督和政协民主监督，人大议案建议、政协提案办复率均达到100%，提请人大审议通过地方性法规6件，制定政府规章、规范性文件16件，修订出台《政府工作规则》及《常务会议规则》。坚决落实中央“八项规定”和区市若干规定，大幅精减节庆论坛展会，撤销检查评比表彰活动54项，“三公”经费同比下降18%。全面完成市及县（市）区两级政府性债务审计，加大效能监察和政务公开，完善问责系列制度，加强勤政警示教育，在全区率先推出电视问政，督促解决道路交通、食品安全等1100多个群众反映的突出问题，机关和干部作风进一步好转，政府公信力和执行力不断提高。

　　各位代表，刚刚过去的一年，全市干部群众团结一心，拼搏奋进，攻坚克难，共同创造了银川综合保税区、滨河新区、阅海湾中央商务区建设，以及道路畅通、老旧小区、棚户区改造、人才科技大厦封顶、银川舰回家等多个“银川速度”，丰富了“贺兰岿然、长河不息”的银川精神，展现了银川人“敢想敢干、敢试敢闯”的时代风貌，推动全市经济社会发展取得了新的可喜成绩。奋斗凝聚汗水，成绩来之不易。这是自治区党委、政府和市委正确领导的结果，是市人大、市政协关心支持的结果，是全市人民共同努力的结果。在此，我代表市人民政府，向辛勤奋战在各条战线的广大干部群众，向给予政府工作大力支持的人大代表、政协委员、各民主党派、工商联、无党派人士和人民团体，向驻银部队指战员、武警官兵、公安民警，向关心支持银川发展的社会各界朋友，表示衷心的感谢和崇高的敬意！

　　在肯定成绩的同时，我们也清醒地认识到，经济总量不大、发展不足仍是我们最大的市情，银川改革发展还面临不少困难和挑战，主要表现在：一是产业转型升级任务艰巨，战略性新兴产业和现代服务业支撑作用不足，科技创新能力不强，节能降耗形势严峻。二是城乡居民收入水平不高，物价上涨压力较大；三是城镇化建设投融资瓶颈亟待破解，城市规划和精细化管理水平有待提高；四是政府职能要进一步转变，制约发展的体制机制需进一步理顺。对此，我们将采取有力措施，认真加以解决，努力把政府工作做好，让人民满意。

　　二、2014年的主要工作

　　2014年是全面深化改革的开局之年，也是完成“十二五”规划的关键一年。做好今年的工作，对于在新形势、新起点上推进银川改革发展，为率先建成全面小康社会奠定更加坚实的基础，具有重大意义。市委十三届三次全会全面分析、部署了今年的形势与任务，提出要抓住深化改革、扩大开放、新型城镇化“三大机遇”，认清面临的加快发展与转型升级、经济增长与节能减排、推进城镇化建设与控制债务风险等重大挑战，处理好GDP增长与持续健康发展、发展速度与发展质量、全面发展与重点突破等重大关系，落实好“2258”工作思路等“七个坚定不移”，全面推动银川经济社会跨越式发展。我们要深入贯彻落实好自治区、市委的决策部署和具体要求，坚持数量与质量并重，突出质量；坚持招商引资与招才引智并重，突出招才引智；坚持银川速度与银川效率并重，突出银川效率，进一步解放思想、坚定信心、抓住机遇、乘势而上，以更大的决心、更实的举措，推动银川率先发展、科学发展、跨越发展。

　　政府工作总的要求是：全面贯彻落实党的十八大及十八届二中、三中全会、自治区十一届三次全会和中央、自治区经济工作会议精神，坚持“稳中求进”总基调，坚持开放内涵式发展，抢抓“两区”建设、“两大战略”机遇，深入实施“2258”工作思路，把改革创新贯穿于经济社会发展各个领域各个环节，加快产业转型升级，加快现代化国际化进程，加快“两个最适宜”城市建设，加快民生改善，加快生态文明建设，着力提高经济发展质量，着力提高城市品位，着力增强社会活力，着力增强群众幸福感，全面建设开放银川、富裕银川、和谐银川、美丽银川，努力开创科学发展、跨越发展新局面。

　　主要预期目标是：地区生产总值增长11%左右，地方公共财政预算收入增长14%以上，全社会固定资产投资增长25%以上，社会消费品零售总额增长13%以上，外贸进出口总额增长15%以上，城镇居民人均可支配收入增长11%左右，农民人均纯收入增长12%左右，城镇登记失业率控制在4.5%以内，居民消费价格总水平涨幅控制在3.5%左右，人口自然增长率控制在8‰以内，单位地区生产总值能耗下降、主要污染物减排完成自治区下达的控制指标。

　　今年重点抓好以下几个方面的工作：

　　（一）突出产业转型，打造银川经济升级版。要统筹推进稳增长、调结构、促转型，做优做强工业，做特做精农业，做活做大服务业，在产业结构调整上迈出新步伐。

　　大力推进新型工业化。深入实施以战略性新兴产业为突破的工业振兴工程，实施重点工业项目320个，工业投资增长20%以上，力争规模以上工业增加值增长13%。做大园区经济。放大园区带动和集聚效应，提升产业配套能力，打造产业集群。把宁东基地作为全市产业发展的基础，加快延伸能源化工产业链条，抓好宝丰煤制烯烃、宝塔石化120万吨PTA等重大项目。把银川经济技术开发区作为引领产业转型的龙头，加快建设银川iBi育成中心，培育发展新一代互联网信息技术、大数据、云计算、“2.5产业”等新经济业态。把宁夏生态纺织产业示范园、银川高新技术产业开发区作为引领轻工业发展的载体，完善园区道路、污水处理等基础设施配套，加快培育羊绒纺织产业集群，打造中国羊绒纺织之都和国家级生态纺织产业基地。出台相关配套政策，支持西夏区实施国家老工业基地调整改造规划。壮大特色产业。大力发展先进装备制造业，以西轴高端轴承产业园、巨能机器人等项目为抓手，提高装备制造业高端化、智能化、信息化水平。大力发展新材料产业，以西安隆基硅、佳晶蓝宝石等项目为重点，打造亚洲最大的新材料生产基地。大力发展生态纺织产业，力促如意集团年产30万吨如意纺高档纱线、中银绒业500万件羊绒衫等项目建成投产，建设灵武羊绒电子交易中心，推进骨干企业品牌、技术、资本国际化。大力发展高端葡萄酒及酒庄产业，加快张裕、中粮、德龙等优质葡萄示范基地及葡萄酒项目建设，打造葡萄种植、加工、展示交易、工业旅游全产业链。大力发展信息产业，推进国家信息消费试点城市建设，支持经济技术开发区加快大数据的开发与应用，抓好西部工业云谷、3D打印技术等前瞻性项目，促进“两化”深度融合。推进企业技术创新和节能降耗。加快技术进步，支持企业产品创新、品牌创新和商业模式创新。打造企业成长梯队，培育一批“专、精、特、新”中小微企业。实施重点技改项目80个，改造提升发酵及生物制药等传统产业。健全重点用能企业监测管理和预警调控机制，推行企业能耗对标达标、能源审计和电力需求侧管理。加强项目节能评估，坚决淘汰铁合金、水泥等落后产能，大力发展循环经济，培育发展节能环保产业。

　　加快推进农业现代化。以农民增收为核心，突出生态、安全、优质、高效，大力发展“三精”农业，加快创建国家现代农业示范区和农产品质量安全区，农业总产值增长5%以上。打造现代农业产业基地。加大政策扶持和引导力度，推进区域化布局、规模化经营、标准化生产，推广应用新品种、新技术、新装备，新建改造30个农业科技示范园区（基地），壮大设施园艺、奶产业、有机大米、适水产业等优势特色产业，新增蔬菜种植面积2万亩、有机水稻1万亩，奶牛存栏达到17万头。构建新型农业经营体系。扶持壮大一批龙头企业、流通主体，加快培育一批专业大户、农民专业合作社、家庭农场，推进多种形式规模经营，完善利益联结和分配机制，提高农民在农业产业化过程中的收益。完善农产品质量检测、动物疫病防控体系，完成10个绿色有机农产品认证认定，打造一批特色品牌，力争更多的农产品打入国内外市场。提升农业综合生产能力。落实支农惠农政策，严守耕地红线，集中连片开展农田水利建设工程，创建高标准农田60万亩，改造中低产田30万亩，新增高效节水示范园区10万亩。完善农业科技服务体系，加强基层农技骨干和农村实用人才培训，培养职业农民。建设一批农业机械化示范园，主要农作物机械化水平达到91%。

　　加快发展现代服务业。抓好国家服务业综合改革试点，推动服务业发展提速、比重提高、层次提升，打造现代服务业示范区，服务业增加值增长9%以上。推动商贸服务业升级。抓好亘元万豪、永泰城、大阅城等十大五星级酒店、十大商业综合体建设，放大万达广场、奥特莱斯名品折扣城品牌效益。加快特色商业街区和社区商业网点建设，大力发展电子商务、连锁、快递等业态，培育新兴消费，打造教育、医疗、购物、旅游在银川品牌，促进消费结构升级。完善现代物流体系。推进全国流通领域现代物流示范城市提档升级，加快公铁物流中心、润恒农产品物流产业园、国际农机汽车城等物流园区建设运营，建设银川国际航空物流中心，培育壮大物流骨干企业，提高物流信息化水平。大力培育会展经济。实施银川国际会展中心二期建设，办好中国西部（银川）房车博览会、国际羊绒暨服饰博览会等品牌展会，培育引进专业会展公司，提高会展配套服务能力。实施“金融强市”战略。争取成立金融工作局，大力引进国内外金融机构，培育银行、证券和保险等现代金融业态，着力打造区域性金融中心、结算中心。创建西部（银川）担保集团，完善担保体系，解决中小企业贷款难、担保难问题。加强上市后备企业培育、储备，支持企业在资本市场直接融资，拓宽企业融资渠道。引入专业化农业保险公司，大力发展村镇银行、农村小额贷款公司、农民资金互助合作组织，构建农村金融体系。推进“诚信银川”建设，建立健全社会征信体系，优化金融生态环境。推进文化旅游融合发展。提升西夏王陵、贺兰山岩画、镇北堡影城、水洞沟等景区功能和服务水平。加快滨河新区全域5A级景区建设，推进兵沟自驾车基地、国际游客集散中心、军事文化博览园二期、永宁国际综合旅游度假区等重点项目，抓好西夏陵申遗及国家考古遗址公园、贺兰山东麓葡萄文化旅游带建设。开发精品线路，打响旅游品牌，全年接待游客800万人次，实现旅游总收入78亿元。

　　（二）突出改革创新，增强发展内生动力。要坚持以改革促发展，激发市场活力，增强发展方式转变的内生动力，努力推动改革实现新突破、创新取得新进展。

　　抓好重点领域改革。深化财政管理体制改革。加强和改进预算管理，推行全过程预算绩效评价。规范市及县（市）区政府性债务管理。进一步明确市与辖区两级事权和支出责任。创新财政扶持产业发展模式，整合专项资金，设立产业转型基金，引入竞争性申请项目资金机制，提高财政资金使用效益。放宽市场准入。充分发挥市场在资源配置中的决定性作用。健全国有资本经营预算制度和绩效考核评价体系，完善现代企业制度。每年统筹安排财政资金5亿元扶持非公有制经济发展，支持引导非公企业和民间投资进入基础设施、公用事业、保障性住房、社会事业等领域，建立投资回报补偿机制。加大中小微企业扶持力度，把本地名优产品优先列入政府采购。创新投融资模式。盘活政府经营性资产和事业单位资产，做实滨河新区、综保区、城投等投资公司，组建住房、体育、文化旅游等融资平台，发行城投债20亿元，提高市政基础设施和城镇化建设投融资能力，缓解财政收支压力。稳步推进事业单位分类改革。全面推进事业单位法人治理结构改革试点，逐步建立功能明确、治理完善、运行高效、监管有力的事业单位管理体制和运行机制。加快农村集体产权制度改革。健全集约节约用地制度，加强土地批后监管。推进农村土地、房屋确权登记，设立农村产权交易中心，开展土地流转信托试点，探索农村资产资本化、证券化路径，进一步壮大村集体经济。

　　实施创新驱动战略。加快创新型银川建设。发挥银川科技园科技研发、产业创新的平台作用，加快研发大楼、人才公寓、高新技术产业孵化园建设，加强与国家大院大所合作，抓好中科院科技创新与产业育成中心、宁东科技园等“园中园”建设。围绕产业转型升级，实施共性关键技术研发和科技成果转化项目80个。突出企业主体，推进产学研协同创新，搭建技术创新平台，新培育高新技术企业10家，国家级、自治区级企业技术中心2家。加强知识产权运用和保护，提高专利申请质量，发明专利申请量占专利申请比重达到30%以上。建立企业科技创新后补助机制，鼓励引导企业加大科技投入。加快“人才特区”建设。坚持人才优先发展，突出“招才引智”，加大柔性引才力度，着力引进高科技领军人才、高技能人才和外经外贸人才等高层次紧缺人才，注重用好用活现有人才。加强与国内、区内知名高校、科研院所合作，鼓励各类人才带项目、带技术、带团队在银创新创业。建立以实绩为导向的科技人才评价激励保障机制，打造西部人才高地。加快“智慧银川”建设，抓好国家“智慧城市”建设试点，引入中兴集团，大力推进公共信息平台、智慧政务、智慧民生等重点工程。

　　（三）突出开放带动，加快建设试验区核心区。要围绕“引领全区、辐射西北、融入世界”的定位，按照自治区建设丝绸之路经济带战略支点的部署，大力推进“两区”建设，打造试验区核心区，开创开放型经济新格局。

　　构建开放战略载体。力促滨河新区出形象。坚持“四城”战略定位，突出国际化、现代化、高端化，打造“生态田园城”。高标准完成新区总体规划、专项规划及控制性详规，加强与临港经济区、综合保税区、河东国际机场区域一体化联动发展，推进与鄂托克前旗上海庙跨区域合作。加快推进203省道辅线、兵沟等3座黄河大桥及连接线和机场三期等重大工程，加快实施国际医疗城、科教城、养生新城、信息产业园等重点项目，建成污水处理厂、滨河生态文化园、黄河外滩公园景观栈道等项目，促成绿地空港城、天山海世界等大项目落地，力争新区在管理运营、基础设施、生态绿化、高端产业培育等方面实现新突破。力促综合保税区快见效。坚持建设、运营、见效并举，逐步启动二期建设，推进国际贸易服务中心、研发检测中心和生活配套区建设。抓好一期入园、注册企业（项目）建设运营，重点引进培育轻纺、葡萄酒、通用航空、融资租赁等主导产业和项目。用好跨境贸易电子商务试点机遇，争取阿里巴巴等电商企业落户，建设线上线下全方位跨境贸易电子商务产业集群。探索建立“一区多园”模式，加快自由贸易园区申报步伐。力争年内外贸进出口额突破13亿美元，再造一个银川外贸，实现运营见效全国最快的目标。力促阅海湾中央商务区早成型。坚持建设、招商、运营并重，优化商务区规划和城市设计，完善基础设施配套，稳步推进阅海湾经济区建设。加快推进绿地“双子塔”、新华联广场等32个重点项目，大力发展总部经济、金融服务等高端商务，争取韩国在银设立领事机构，建成国际化、现代化示范区。

　　扩大开放合作。构建开放通道。利用第五航权开放，开辟银川至中亚、中东、东南亚等国际航线，推进银川至首尔等国际航班常态化运行，争取“落地签”、“过境签”和外国人居住证等促进出入境便利化的政策。完善银川陆港口岸功能，发展公铁联运、铁海联运，加强与内蒙古策克、新疆霍尔果斯等口岸及天津、连云港等港口的合作，加快形成向西出境、向东出海的便捷通道。拓展开放平台。推进与天津、杭州等发达地区和毗邻地区的经济合作，深化与潮商、台商的务实合作，承接东部沿海地区加工贸易链条延伸。加快发展外向型经济。制定促进外贸发展的实施意见，依托国家级外贸转型升级示范基地，吸引国外资金和国内民营资本；支持有条件的企业赴外投资，设立特色产品销售中心，拓展中亚、北非等新兴市场。完善选商引资机制，紧盯战略性新兴产业，大力引进国际国内500强企业和战略投资者，加强签约项目的跟踪落实，提高招商引资的质量和实效，力争全年招商引资实际到位资金突破430亿元。

　　（四）突出城乡统筹，推进新型城镇化。要抓住宁夏沿黄等10个区域性城市群列入国家城镇化规划的战略机遇，按照自治区打造“大银川都市区”的要求，加快新型城镇化步伐，争创联合国人居奖。

　　完善规划体系。坚持“全域银川、城乡统筹”理念，完成城市发展战略策划、产城一体化规划、生态规划编制，调整修编城市总体规划。优化重点区域、重要节点城市设计，严格控制老城区建筑（住宅）高度，突出城市“三大特色”，保持历史文化名城风貌。优化城市空间布局，提升永宁、贺兰与市区“同城化”发展水平，加快灵武市重心北扩，构建城乡融合、多级支撑、组团发展的新型城镇化格局，争取列入国家新型城镇化试点。加强城乡规划管理，在两县一市设立规划分局，深入开展违法建设专项整治行动，切实维护规划的严肃性。

　　优化城市功能。充分发挥区域中心城市和沿黄城市带核心城市带动作用，着力在功能完善、品位提升、形象塑造上做好示范和引领。深入推进棚户区改造工程，高起点、高标准建设安置区，回迁安置群众1.7万户、183万平方米，改造城市棚户区2.7万套。大力实施道路畅通工程，新建、改造道路18条，实施一批道路微循环和慢行交通工程。优先发展公共交通，加快公交线网向新建小区和农村延伸，推进公交场站、换乘枢纽建设，提高公交运营服务水平，创建公交都市示范城市。完善智能交通系统，优化交通组织和路口渠化、信号灯配时，大力倡导文明交通、绿色出行，有效治理交通拥堵。加强城市基础设施建设，优化城市地下管网设计功能，实施建成区老旧管网升级改造工程，改造供水管网41公里、燃气管网69公里，改造集中供热面积77.66万平方米，完成既有建筑节能改造200万平方米，推行热计量收费100万平方米。推进污水处理扩容升级，新建第七、第九污水处理厂，实施贺兰山路等城市防洪排水工程，全面提升基础设施配套水平。

　　精细管理城市。深入开展城市精细化管理，制定城市管理规划，提高管理标准，全面推进城乡环境综合整治。加强占道经营、户外广告、“渣土车”标准化、规范化管理。推进垃圾分类处理和资源化利用，建成生活垃圾焚烧发电及餐厨垃圾集中处理项目。稳步推进环卫作业专业化、市场化，逐步实行作业和管理分离，提高机械化清扫率。完善“大城管”体制机制，提高“数字城管”运行效率，实现全方位、精细化、实时有效管理。深化文明细节专项行动，扎实开展群众性精神文明创建活动，着力塑造“美丽心灵”，提升市民素质，培育城市人文精神。

　　建设“美丽乡村”。建好镇北堡、通贵等6个重点小城镇，促进城镇功能与产业定位的有机融合，推动城市基础设施向农村延伸、公共服务向农村覆盖、现代文明向农村辐射。抓好绿色中心村试点，推行农村生活垃圾“户集、村收、乡运、县处理”，建设人口集中、设施齐全、环境优美、农业现代化的新农村。完善农民变市民的政策措施，统筹户籍、就业、教育、医疗、社会保障等政策，实现城镇基本公共服务常住人口全覆盖，有序推进农业转移人口市民化。

　　（五）突出绿色发展，加强生态环境治理。要始终坚持生态优先、保护环境，做好“绿化、水系、空气”三篇文章，打造“碧水蓝天、明媚银川”新名片，加快创建国家生态园林城市。

　　提升生态品位。坚持扩大绿量，提升档次，打造精品，新建完善园林绿化面积500公顷，造林5700公顷。继续推进大整治大绿化工程，抓好滨河新区大环境绿化、唐徕公园六期整治和“绿博园”建设，实施友爱中心路、正源街等主要道路景观林带及配套绿化工程，加强银西生态防护林带建设，构建高密度、大绿量、多色彩的城市“绿网”。完善城市水系，实施绕城水系连通、爱伊河景观提升、阅海环湖湿地改造等工程，加强湖泊湿地生态修复、保护和利用。新建10个小微公园，做好街头绿地、小游园的精品化改造，打造“一街一品，一街一景”，让市民亲水近绿、亲近自然。

　　强化环境保护。加快生态保护立法，划定生态红线，严格“绿线”控制，用制度保护生态环境。建立环境保护绩效考核、责任追究、社会监督等机制，严格环保准入和污染物排放标准，对环境违法行为“零容忍”。制定全市沟渠综合整治规划，实施二排沟、四二干沟城市段综合治理，加强贺兰山东麓等水源地保护，抓好农村面源污染治理。深入实施“蓝天工程”，加强环境空气质量监测分析和联防联控，抓好工业废气、机动车尾气、燃煤锅炉、施工扬尘等污染源专项整治，打造空气质量西部最优“金字招牌”。

　　（六）突出民生改善，大力发展社会事业。要坚持民生导向、富民优先，把新增财力主要用于保障改善民生，加快完善基本公共服务体系，加快形成科学有效的社会治理机制，让城乡居民的幸福感进一步提高。

　　加大民生工作力度。坚持不懈地办好群众最关心、最需要的事情，继续为民办10件重点实事。建立健全创业就业公共服务体系，深入推进创业孵化基地建设，完善人力资源市场功能，实施创业就业培训1万人以上，统筹做好高校毕业生、城镇困难人员和农村转移劳动力就业，新增城镇就业2.5万人。持续推进社会保险扩面提标，统筹城乡居民养老保险和医疗保险，建立完善城乡居民大病保险制度。继续提高城乡低保标准和优抚对象补助水平，实施殡葬惠民政策，扩大社会救助覆盖面。加快社会养老服务体系建设，支持社会力量兴办养老服务业，提升社区养老服务水平，健全农村留守儿童、妇女、老年人和残疾人关爱服务体系。实行公租房与廉租房“合二为一”，新建各类保障性安居工程9000套，实现低收入住房困难家庭应保尽保。优化土地、住房供应结构，促进房地产市场平稳健康发展。保持物价基本稳定，建设蔬菜生产基地3~6个，推行蔬菜订单直销模式，升级改造社区蔬菜直销店50个，减少流通环节，加大商业储备投放，增强物价调控能力。创新扶贫开发模式，坚持扶贫与扶“志”（志气、智力、素质）相结合，突出抓好生态移民安置、主导产业培育、创业就业培训、劳动密集型企业引进等工作，高标准推进闽宁镇及“一城两园”建设，力争移民地区人均纯收入突破8000元、增长20%以上。

　　加快社会事业发展。推进教育优先发展。新建续建中小学、幼儿园30所。推进公建民办、民办公助，扩大公益性、普惠性学前教育资源。持续推进优质教育扩面提升工程，组建银川六中、九中等学校发展共同体，促进城乡、县域内义务教育均衡发展。推进普通高中教育多样化、特色化办学，完成宁夏幼儿师范学校升格。创新职业教育办学和公共实训机制，启动建设滨河新区教育园区，打造西部职业教育高地。抓好国家中小学教育质量综合评价改革试点，实施“梯级”名师培养工程，推行校长、教师交流轮岗，破解择校难题，让教育公平惠及更多家庭。提高医疗卫生服务水平。加快公立医院改革，完善公立医院绩效评价和财政补贴机制。允许民办医疗机构纳入医保定点范围，鼓励支持民营医院建设和发展。完善基层医疗卫生服务体系，推进分级诊疗，引导群众小病进社区，大病进医院。推进中俄韩医疗合作，启动中航公司造血干细胞库、细胞治疗中心项目，让群众享受到高水平的医疗服务。实施“健康银川”行动，加强妇幼保健和公共卫生工作。适时启动“单独二孩”政策，促进人口长期均衡发展。完善公共文化服务体系。实施文化惠民工程，健全完善四级公共文化服务网络，深入开展“书香银川·银川书香”活动，推动新闻出版、广播影视、文学艺术、档案事业繁荣发展。大力发展文化创意、动漫游戏等新兴文化产业。加快建设运动休闲城市，推广“人人运动计划”，承办自治区第十四届运动会，提高贺兰山体育场等文体设施运营管理水平。

　　创新社会治理。加强基层社会服务治理体系建设，扩大网格化、智能化社区试点。抓好流动人口、特殊人群和虚拟社会的管理服务。培育扶持行业商（协）会等社会组织，大力发展志愿服务组织，支持工会、共青团、妇联等群团组织发挥作用。深化平安创建，推进“六五”普法教育，扩大法律援助覆盖面，完善“大调解”工作机制，创新化解社会矛盾机制，完善立体化社会治安防控体系，加大治安重点地区整治力度，增强群众安全感。加强从田间到餐桌的全程监管，让群众吃得放心。严厉打击食品药品安全违法违规行为。严格落实安全生产政府监管、企业主体责任，加强危险化学品、道路交通、消防、建筑施工、地下管网等重点领域安全隐患排查治理，有效预防和减少重特大事故。加强防灾减灾、应急管理工作。积极创建双拥模范城“八连冠”，加强国防动员、民兵预备役和人防建设，推动军民深度融合发展。创新开展民族团结进步创建活动，依法加强宗教事务管理，倍加珍惜和维护民族团结、宗教和顺、社会稳定的大好局面。

　　（七）坚持为民务实清廉，建设人民满意政府。我们要按照全面深化改革的新要求，顺应人民对政府工作的新期待，认真开展党的群众路线教育实践活动，进一步转变职能、优化环境、改进作风、提高效能，让社会和群众看到实实在在的变化。

　　转变政府职能。完成县（市）区政府机构改革，规范机构设置。试行权力清单制度，厘清政府与市场以及社会的边界，强化政府公共服务、市场监管、社会管理、环境保护等职责。建立政府向社会力量购买公共服务机制，制定购买目录，在学前教育、基层医疗卫生、城乡社区服务等领域扩大试点。深化行政审批制度改革，建立行政审批事项“负面清单”，实行“三个一律”、“三个50%”，大力推进“三减两提高”。启用新建市民大厅，审批服务“一门受理、多证联办”，推行网上审批、电子监察，打造“两优”环境示范区。

　　推进依法行政。完善重大事项集体决策、专家咨询、社会公示、听证评估等制度。严格落实执法责任制，强化执法监督，加强行政复议工作，全面推进法治政府建设。主动接受人大依法监督和政协民主监督，提高人大代表建议、意见和政协委员提案办理质量。大力推行政务公开，推动电视、网络问政向电视、网络施政转变，让群众更好地了解政府、监督政府。

　　改进工作作风。大力改进文风会风，文件和会议数量再压缩20%以上，严控各类庆典表彰和评比达标活动。从政府领导班子做起，带头转变工作作风，带头深入基层调查研究，带头密切联系群众，带头解决实际问题。推行政府绩效管理，完善重点工作公开承诺、目标管理和督查考核机制。开展“正风提效促跨越实现银川速度向银川效率提升”活动，加大对重点工程、重大项目、民生工作的督查力度，强化行政问责，着力整治公务人员庸懒散慢等不良现象，提高政府效能和执行力。

　　做到廉洁从政。认真执行《党政机关厉行节约反对浪费条例》，推进财政预算、决算公开，严格公务接待、公务用车、办公用房标准，严控公务出国（境），“三公”经费再压缩5%。落实党风廉政建设责任制，加强政府内部监督、行政监察和审计监督，抓好公共资源交易、政府投资项目、民生资金等重点领域的监管。严查各类违纪违法案件，着力解决群众反映突出的政风行风问题，以政府勤政廉政实际成效取信于民。

　　各位代表，美好前景催人奋进，新的征程任重道远。让我们在自治区党委、政府和市委的坚强领导下，紧紧依靠全市人民，团结拼搏，锐意进取，全面建设开放银川、富裕银川、和谐银川、美丽银川，为率先建成全面小康社会、实现跨越式发展而努力奋斗！
